# Synthesis, structural characterization, and optical properties of benzo[*f*]naphtho[2,3-*b*]phosphoindoles

**DOI:** 10.3762/bjoc.17.56

**Published:** 2021-03-05

**Authors:** Mio Matsumura, Takahiro Teramoto, Masato Kawakubo, Masatoshi Kawahata, Yuki Murata, Kentaro Yamaguchi, Masanobu Uchiyama, Shuji Yasuike

**Affiliations:** 1 School of Pharmaceutical Sciences, Aichi Gakuin University, 1-100 Kusumoto-cho, Chikusa-ku, Nagoya 464-8650, Japan; 2 Pharmaceutical Sciences at Kagawa Campus, Tokushima Bunri University, 1314-1 Shido, Sanuki, Kagawa 769-2193, Japan; 3 present address: Showa Pharmaceutical University, 3-3165 Higashi-Tamagawagakuen, Machida, Tokyo 194-8543, Japan; 4 Cluster of Pioneering Research (CPR), Advanced Elements Chemistry Laboratory, RIKEN, 2-1 Hirosawa, Wako, Saitama 351-0198, Japan; 5 Graduate School of Pharmaceutical Sciences, The University of Tokyo, 7-3-1 Hongo, Bunkyo-ku, Tokyo 113-0033, Japan; 6 Research Initiative for Supra-Materials (RISM), Shinshu University, 3-15-1 Tokida, Ueda, Nagano 386-8567, Japan

**Keywords:** benzo[*f*]naphtho[2,3-*b*]phosphoindole, molecular structure, optical property, phosphorus

## Abstract

Phosphole-fused π-conjugated acenes have been attracting interest because of the attractive features of the phosphole moiety, such as fluorescence and chemically modifiable properties. Herein, 6-phenyl-6*H*-benzo[*f*]naphtho[2,3-*b*]phosphoindole was prepared by reacting dichlorophenylphosphine with a dilithium intermediate derived from 3,3′-dibromo-2,2′-binaphthyl. Various derivatives, such as a phospholium salt and a borane–phosphole complex with functional groups on the phosphorus atom were synthesized using the obtained phosphole as a common starting material. Single-crystal X-ray analysis of the parent benzo[*f*]naphtho[2,3-*b*]phosphoindole revealed that the pentacyclic ring is almost planar. Fluorescence spectroscopy data showed that the phosphole derivatives, such as phosphine oxide and the phospholium salt and borane complex exhibited photoluminescence in chloroform.

## Introduction

Phosphole-based heteroacenes are attracting increasing interest in various fields, such as organic synthesis, structural chemistry, and materials science [1–4]. The phosphorus atom of trivalent phosphorus compounds has a high chemical reactivity. Therefore, this phosphorus center can be easily chemically modified and converted to phosphole derivatives with different electronic properties by reactions such as oxidation, alkylation, and coordination to a Lewis acid [1–8].

**Figure 1:**
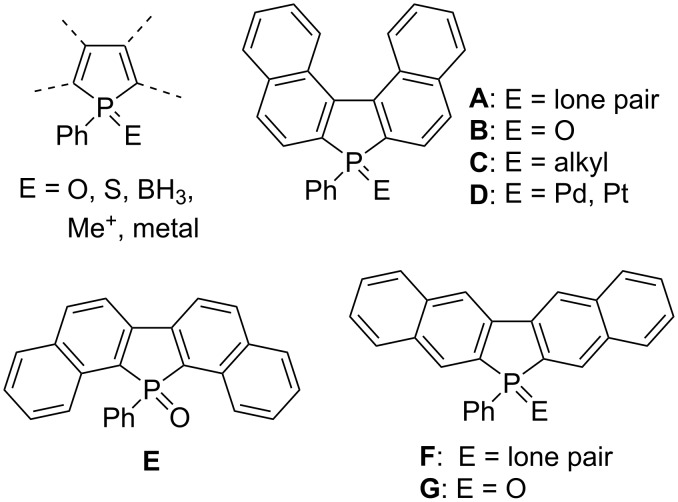
Benzonaphthophosphindoles.

Theoretically, pentacyclic benzonaphthophosphindole contains six structural isomers, in which the position of the fused benzene rings is different; of these, three are shown in Figure 1. The synthesis, crystal structure, and dynamic behavior of benzo[*e*]naphtho[2,1-*b*]phosphindole (**A**) with the *C*_2_ symmetry axis on the binaphthyl skeleton have been reported [9–11]. Synthetic approaches for phosphine oxide **B** [12–14], alkylated products **C** [15], and transition metal complexes **D** [16–17] have also been developed. However, for isomers **E** [18–19] and **G** [20], only the synthetic method for pentavalent phosphine oxides has been reported. To the best of our knowledge, the synthesis and derivatization of trivalent phosphole **F** and the optical properties have not been clarified. The systematic knowledge of the properties of a new family of fused phospholes is valuable for the design of new types of functional π-electron-containing materials. This paper presents the synthesis, molecular structure, and optical properties of 6-phenyl-6*H*-benzo[*f*]naphtho[2,3-*b*]phosphoindole (**F**) and the derivatives in which the phosphorus atom is chemically modified, such as a phospholium salt and the borane–phosphine complex.

## Results and Discussion

**Scheme 1:**
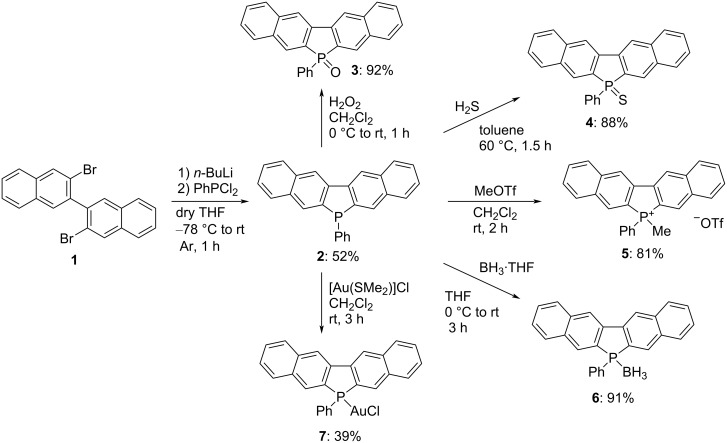
Synthesis of benzo[*f*]naphtho[2,3-*b*]phosphoindoles.

Treatment of 3,3′-dibromo-2,2′-binaphthyl (**1**) [21] with *n*-butyllithium in dry THF at −78 °C and subsequently with dichlorophenylphosphine resulted in ring closure, affording the desired product containing 6-phenyl-6*H*-benzo[*f*]naphtho[2,3-*b*]phosphoindole (**2**) in 52% yield via the 3,3′-dilithio-2,2′-binaphthyl intermediate. The chemical modification of the phosphorus atom of **2** was carried out; the results are shown in Scheme 1. The reaction of **2** with hydrogen peroxide or elemental sulfur afforded the corresponding phosphine oxide **3** and sulfide **4** in 92% and 88% yield, respectively. Treatment of **2** with methyl triflate afforded phospholium triflate **5** in 81% yield. The reaction of **2** with borane in THF formed a borane complex **6** in 91% yield, and the reaction with chloro(dimethyl sulfide)gold afforded the gold complex **7** in 39% yield.

**Figure 2:**
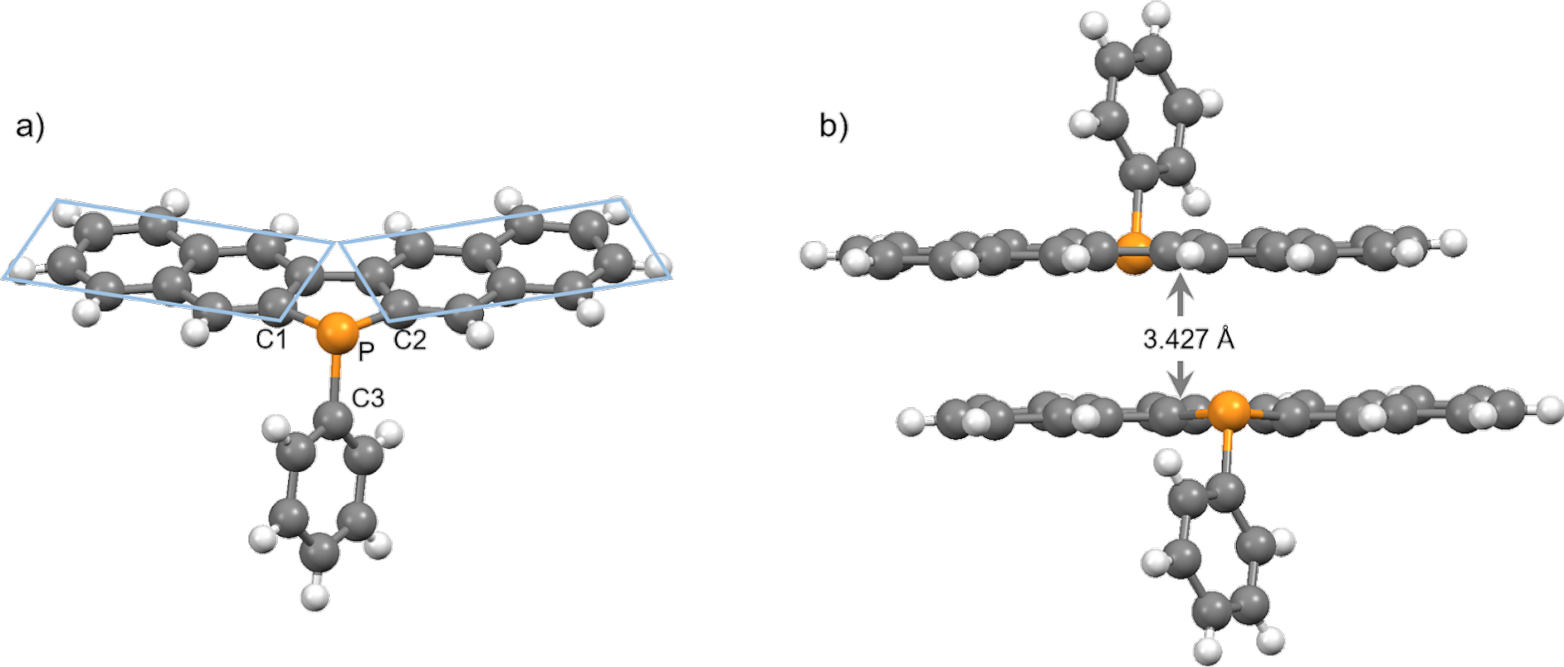
Crystal structure of **2**: different views.

The molecular structures of the synthesized phospholes **2**−**7** were confirmed by spectral analyses (^1^H, ^13^C, and ^31^P NMR as well as MS and IR). All the corresponding aromatic proton and carbon atoms on the two naphthalene rings were equivalent in the ^1^H and ^13^C NMR spectra of phospholes. These results show that all phosphole derivatives had a symmetric structure in solution. The ^31^P NMR spectra of these show the typical low-field shift for P-modified phospholes **3**–**7** (δ = 22.5−39.3 ppm) relative to that of the parent compound **2** (δ = −13.27 ppm). These results suggest that the electron densities of the latter were reduced in comparison to that of **2**. Single crystals of **2** suitable for X-ray analysis were obtained by repeated recrystallization. The molecular structure of **2**, determined through single-crystal X-ray diffraction analysis, is illustrated in Figure 2, and selected geometrical parameters are shown in Table 1. The results revealed that the naphthalene and fused phosphole rings are almost coplanar (mean deviation = 0.030 Å). The angle between each naphthalene ring containing ten carbon atoms is 1.64°, which is smaller than that for group 15 analogs (i.e., *N*-phenyldibenzo[*b*,*h*]carbazole: 4.47° or 2.57° [22], for crystal data, see Figure S2, Supporting Information File 1 and Sb-phenyldinaphtho[2,3-*b*:2′,3′-*d*]stibole: 8.05° [23]). The sum of the bond angles around the phosphorus atom is 295.99°, and hence the phosphorus atom is sp^3^-hybridized and has a trigonal pyramidal geometry. X-ray analysis revealed that the packing structure of **2** had π–π-stacking, with a distance of approximately 3.427 Å between two benzonaphthophosphoindole planes (Figure 2b).

**Table 1:** Selected bond lengths and angles.

bond length (Å)		bond angle (°)	

P–C1	1.8325(13)	C1–P–C2	89.66(6)
P–C2	1.8224(13)	C1–P–C3	104.80(6)
P–C3	1.8375(13)	C2–P–C3	101.53(6)

**Figure 3:**
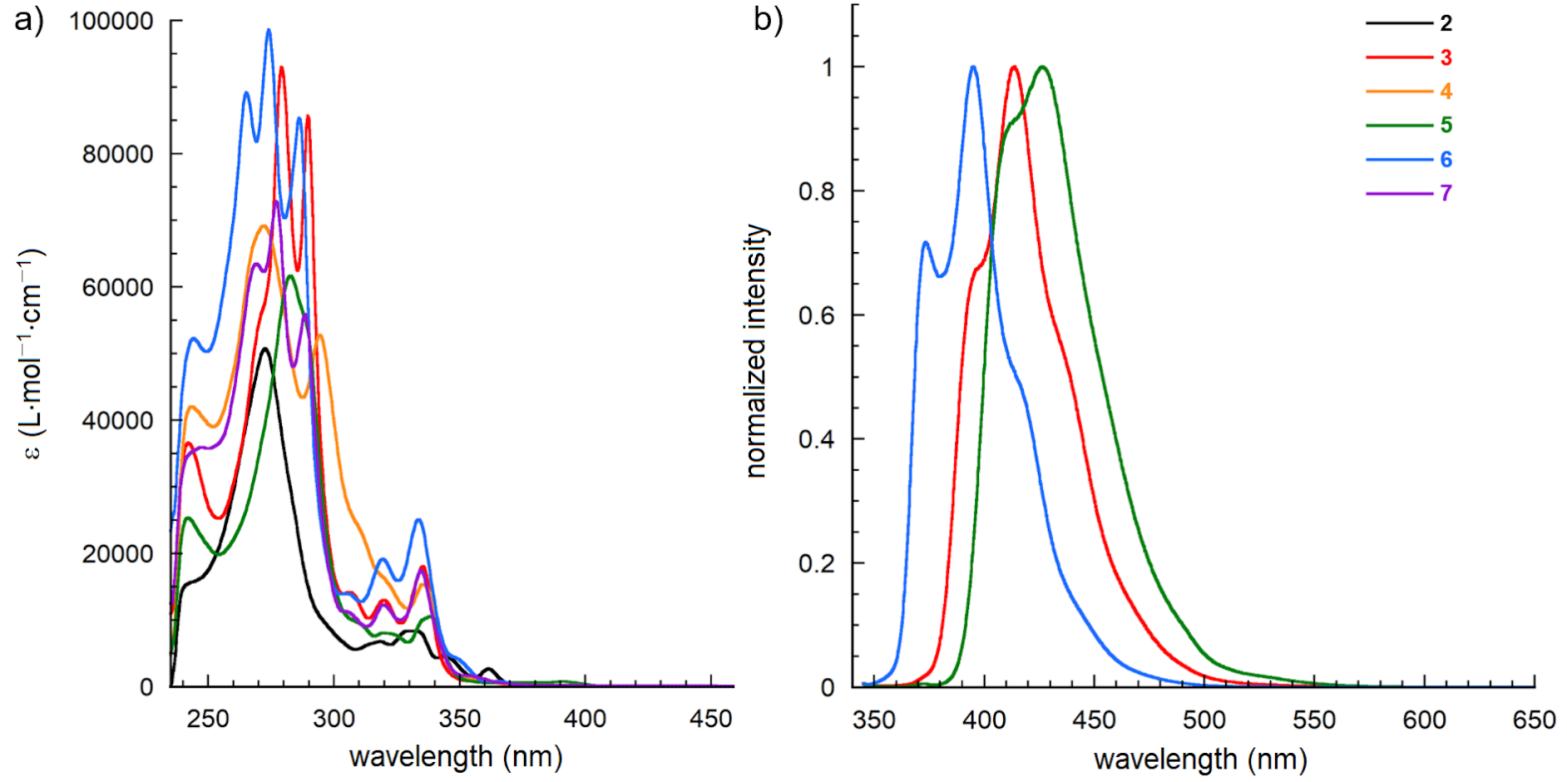
a) Absorption spectra and b) normalized fluorescence spectra for selected compounds in CHCl_3_.

The photophysical properties of the synthesized phospholes were evaluated. The corresponding data are shown in Figure 3 and Table 2. Parent compound **2** shows the absorption maximum (λ_max_) at 362 nm, which is longer than that of 2,2′-binaphthyl (λ_max_ = 300 nm) [24]. The introduction of a phosphorus atom to 2,2′-binaphthyl results in the formation of a bridge that fixes two naphthalene rings in a coplanar axis and forms a heterole-fused system. The λ_max_ values of derivatives **3**–**7** are similar to each other and have a shorter absorption band than that of parent **2**. Furthermore, it is known that λ_max_ of [*n*]helicenes generally has a shorter wavelength than for the corresponding linear poly(acene)s [25]. The λ_max_ of benzo[*e*]naphtho[2,1-*b*]phosphindole (**A**), 358 nm [16], is the same as that of parent **2**. In contrast, the corresponding oxide **B** has a λ_max_ wavelength that is approximately 50 nm longer than that of our oxide **3** [16]. The fluorescence wavelength, including the maximum emission (λ_em_), and the quantum yield depend on the nature of the P-modification. Phosphine oxide **3**, cation **5**, and boron complex **6** emitted blue fluorescence in the visible-light region, with λ_em_ at 395–426 nm (Table 2). P-methylated cation **5** exhibited the longest wavelength and the highest quantum yield.

**Table 2:** Absorption and emissions spectroscopy data.^a^

compound	λ_abs_ (nm) and ε	λ_fl_^b^ (nm)	Φ_fl_^b,c^ (%)

**2**	273 (50800), 362 (2700)	466	0.2
**3**	279 (93000), 336 (18100)	414	8.6
**4**	295 (52800), 336 (15400)	409	0.4
**5**	283 (61700), 339 (10600)	426	25
**6**	274 (98600), 334 (25100)	395	14
**7**	277 (72900), 335 (17500)	406	0.1
**A** [16]	287 (15800), 358 (12600)	—	—
**B** [16]	351 (4000), 387 (5000)	—	—

^a^Measured in CHCl_3_. ^b^Excited at 335 nm. ^c^Measured using anthracene as a standard.

The electrochemical properties of benzonaphthophosphoindoles were investigated by using cyclic voltammetry, and the electrochemical data are summarized in Table 3 and Figure S4, Supporting Information File 1. Parent compound **2** and borane complex **6** showed reversible reduction peaks (*E*_red_ = −1.25 and −1.26 V, respectively). Due to the increased electron–acceptor character of the phosphorus center, P-modification compounds **4** and **5** show a more positive oxidation potential than parent **2** [26]. Unfortunately, the electrochemistry of some compounds could not be determined under the conditions available to us.

**Table 3:** Cyclic voltammetric data^a^ (*E* vs Ag/AgCl) and calculated HOMO and LUMO levels^b^ of phospholes.

compound	*E*_ox_ (V)	*E*_red_ (V)	HOMO (eV)	LUMO (eV)	HOMO–LUMO gap (eV)

**2**	0.99^c^	−1.26^d^	−5.73	−1.78	3.95
**3**	—	—	−6.01	−2.06	3.95
**4**	1.17^c^	—	−5.68	−2.08	3.60
**5**^e^	1.00^c^	—	−8.67	−4.85	3.82
**6**	—	−1.25^d^	−5.99	−2.03	3.96

^a^Measured in DCB with TBAP. ^b^DFT calculation at the level of B3LYP/LanL2DZ. ^c^Irreversible. ^d^Reversible. ^e^Cation part only.

**Figure 4:**
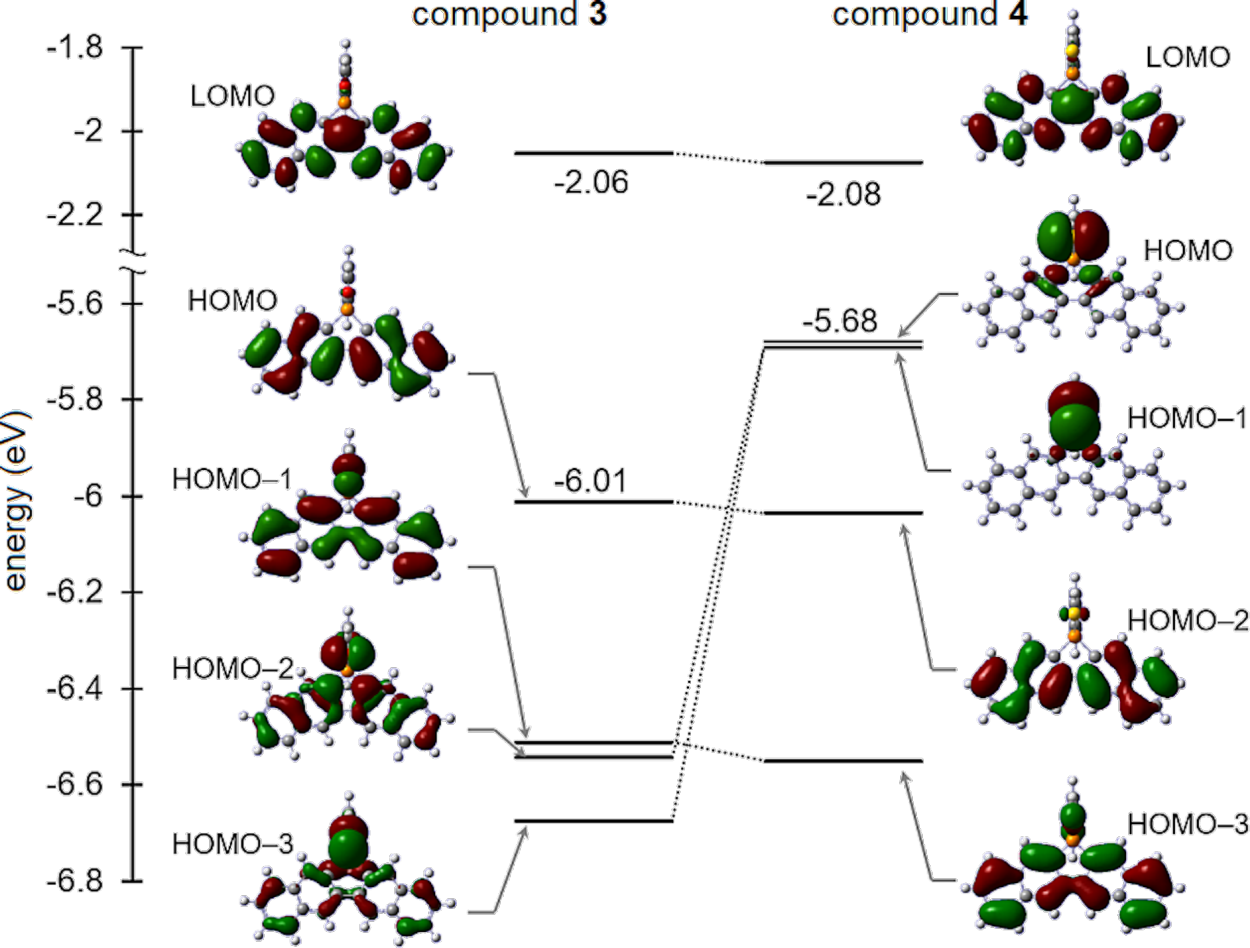
The spatial plots of the HOMO−3 to LUMO of compounds **3** and **4**. The calculations were performed at the level of B3LYP/LanL2DZ.

Therefore, computational investigations are particularly useful for understanding the trends of the electrochemical and photophysical properties of molecular materials. Density functional theory (DFT) calculations [27] were performed at the B3LYP/LanL2DZ level of theory to gain additional understanding of the electronic structures. The HOMO and LUMO energies of the selected compounds are listed in Table 3. The DFT calculations showed that these compounds have HOMO–LUMO gaps of 3.60–3.96 eV. For parent compound **2** and derivatives **3**, **5**, and **6**, the HOMO and LUMO correspond to the π and π* orbitals of the benzonaphthophosphoindole skeleton, respectively (Figure S5, Supporting Information File 1). In contrast, the corresponding π orbitals of phosphole sulfide **4** comprise HOMO−2 energy levels (Figure 4). The HOMO and HOMO−1 levels of oxide **3** are delocalized in the π orbitals of the benzonaphthophosphoindole skeleton, while HOMO−2 and HOMO−3 have a large contribution from the lone-pair and π orbitals. In sulfide **4**, the sulfur analog of the oxide **3**, the HOMO and HOMO−1 energy levels are delocalized in the lone-pair orbitals on the sulfur atom, resulting in considerable destabilization and significantly small HOMO–LUMO gap compared to those of other phospholes **2**, **3**, **5**, and **6**. This result is similar to the reduction potentials of compounds **2** and **6**. According to time-dependent DFT calculations for **4**, the S0 to S1 transitions are mainly dominated by the dipole-forbidden lone pair–π* HOMO–LUMO transitions. This phenomenon may be responsible for the nonfluorescence of **4**. Both the π and π* energy levels in the all-functionalized phosphole derivatives **3**–**6** are lower than those of parent phosphole **2**, owing to the increased electron deficiency of the phosphorus center in the former. In particular, the energy levels in cationic phospholium **5** are significantly stabilized because of the cationic nature of the phosphorus center. These results are consistent with the ^31^P NMR observations discussed above.

## Conclusion

In summary, a novel pentacyclic phosphole, 6-phenyl-6*H*-benzo[*f*]naphtho[2,3-*b*]phosphoindole, was prepared by performing the ring-closure reaction of 3,3′-dilithio-2,2′-binaphthyl with dichlorophenylphosphine. The obtained product was used as a key compound for the chemical modification of the phosphorus atom. X-ray crystal analysis showed that the parent trivalent phosphole has a considerably planar benzonaphthophosphoindole skeleton in its crystal structure. ^1^H and ^13^C NMR observations revealed that all the phospholes obtained in this study had a highly symmetric structure in solution. Fluorescence spectroscopy data showed that the phosphole derivatives, such as a phosphine oxide, phospholium salt, and borane complex, exhibited photoluminescence in chloroform. The π and π* levels in all-functionalized phosphole derivatives are lower than those of the parent phosphole, owing to the increased electron deficiency of the phosphorus center in the former. This electronic nature is supported by the low-field shift of the P-modified derivatives relative to parent phosphole **2** in ^31^P NMR. Further investigation of the design, synthesis, and theoretical and spectroscopic studies of new functional π-electron materials for organic electronics applications is under progress, and the results will be reported in due time.

## Supporting Information

File 1 Further analytical and experimental data.

File 2 X-ray crystal structure of **2**.

File 3 X-ray crystal structure of *N*-phenyldibenzocarbazole.

## References

[1] Romero-Nieto C, Baumgartner T (2013). Synlett.

[2] Ren Y, Baumgartner T (2012). Dalton Trans.

[3] Fukazawa A, Yamaguchi S (2009). Chem – Asian J.

[4] Matano Y, Imahori H (2009). Org Biomol Chem.

[5] Hibner-Kulicka P, Joule J A, Skalik J, Bałczewski P (2017). RSC Adv.

[6] Baumgartner T, Réau R (2006). Chem Rev.

[7] Hissler M, Dyer P W, Réau R (2003). Coord Chem Rev.

[8] Nyulászi L (2001). Chem Rev.

[9] Dore A, Fabbri D, Gladiali S, De Lucchi O (1993). J Chem Soc, Chem Commun.

[10] Watson A A, Willis A C, Wild S B (1993). J Organomet Chem.

[11] Gladiali S, Dore A, Fabbri D, De Lucchi O, Valle G (1994). J Org Chem.

[12] Lian Z, Bhawal B N, Yu P, Morandi B (2017). Science.

[13] Baba K, Masuya Y, Chatani N, Tobisu M (2017). Chem Lett.

[14] Fujimoto H, Kusano M, Kodama T, Tobisu M (2019). Org Lett.

[15] Fabbri D, Gladiali S, De Lucchi O (1994). Synth Commun.

[16] Tani K, Tashiro H, Yoshida M, Yamagata T (1994). J Organomet Chem.

[17] Gladiali S, Fabbri D, Banditelli G, Manassero M, Sansoni M (1994). J Organomet Chem.

[18] Onoda M, Koyanagi Y, Saito H, Bhanuchandra M, Matano Y, Yorimitsu H (2017). Asian J Org Chem.

[19] Nogi K, Yorimitsu H (2017). Chem Commun.

[20] Baba K, Tobisu M, Chatani N (2015). Org Lett.

[21] Motomura T, Nakamura H, Suginome M, Murakami M, Ito Y (2005). Bull Chem Soc Jpn.

[22] Matsumura M, Kawahata M, Muranaka A, Hiraiwa M, Yamaguchi K, Uchiyama M, Yasuike S (2019). Eur J Org Chem.

[23] Matsumura M, Matsuhashi Y, Kawakubo M, Hyodo T, Murata Y, Kawahata M, Yamaguchi K, Yasuike S (2021). Molecules.

[24] Chebaane K, Guyot M (1977). Tetrahedron.

[25] Chen C-F, Shen Y (2017). Helicene Chemistry: From Synthesis to Applications.

[26] Dienes Y, Eggenstein M, Kárpáti T, Sutherland T C, Nyulászi L, Baumgartner T (2008). Chem – Eur J.

[27] (2016). Gaussian 16.

