# Supplementary material for: Synthesis, structural characterization, and optical properties of benzo[f]naphtho[2,3-b]phosphoindoles
Source: Beilstein J Org Chem. 2021 Mar 5;17:671–7. doi: 10.3762/bjoc.17.56 (PMC7940816; doi:10.3762/bjoc.17.56)

BEILSTEIN JOURNAL OF ORGANIC CHEMISTRY

Supporting Information

for

Synthesis, structural characterization, and optical properties of benzo[*f*]naphtho[2,3-*b*]phosphoindoles

Mio Matsumura, Takahiro Teramoto, Masato Kawakubo, Masatoshi Kawahata, Yuki Murata, Kentaro Yamaguchi, Masanobu Uchiyama and Shuji Yasuike

Beilstein J. Org. Chem. **2021,** *17,* 671–677. doi:10.3762/bjoc.17.56

Further analytical and experimental data

License and Terms: This is a supporting information file under the terms of the Creative Commons Attribution License (https://creativecommons.org/licenses/by/4.0). Please note that the reuse, redistribution and reproduction in particular requires that the author(s) and source are credited and that individual graphics may be subject to special legal provisions.
The license is subject to the *Beilstein Journal of Organic Chemistry* terms and conditions: (https://www.beilstein-journals.org/bjoc/terms)

1. General information

Melting point measurements were conducted on a Yanagimoto micro melting point hot-stage apparatus (MP-S3) and reported as uncorrected values. ^{1}H NMR (TMS: δ = 0.00 ppm as an internal standard), ^{13}C NMR ($CDCl_3$: δ = 77.0 or CD_3OD: δ = 49.0 ppm as an internal standard) and ^{31}P NMR (85% H_3PO_4: δ = 0.0 as an external standard) spectra were recorded on JEOL ECZ-400S (for ^{1}H, ^{13}C and ^{31}P NMR, 400, 100 and 161 MHz, respectively) spectrometers. Mass spectra were obtained on a JEOL JMP-DX300 instrument (70 eV, 300 μA). IR spectra were recorded on a FTIR-8400S system from Shimadzu spectrometer and were reported in frequency of absorption (cm^{-1}). Only selected IR absorbencies are reported. UV–vis spectra were recorded at room temperature on a HITACHI U-2800A spectrophotometer (**2**: C = 3.0×10^{-5}, **3**: C = 1.7×10^{-5}, **4**: C = 2.6×10^{-5}, **5**: C = 2.7×10^{-5}, **6**: C = 2.7×10^{-5} and **7**: C = 1.8×10^{-5} M in $CHCl_3$) and fluorescence spectra on a JASCO FP-8300 luminescence spectrometer (**2**: C = 3.4×10^{-6}, **3**: C = 2.2×10^{-6}, **4**: C = 1.4×10^{-6}, **5**: C = 2.1×10^{-6}, **6**: C = 1.5×10^{-6} and **7**: C = 2.6×10^{-6} M in $CHCl_3$). Cyclic voltammograms were recorded at room temperature on HZ-7000 systems from Hokuto Denko (Pt lod as working electrode; in dichlorobenzene solution (≈1.0 mM) with 0.1 M NBu_4ClO_4 as supporting electrolyte; scan rate 100 m·Vs^{-1}; potentials are referred to an Ag/AgCl/KCl 1 M electrode). All chromatographic separations were accomplished with Silica Gel 60N (Kanto Chemical Co., Inc.). Thin-layer chromatography (TLC) was performed using Macherey-Nagel precoated TLC plates Sil G25 UV_{254}.

2. Preparation and characterization of phospholes

6-Phenyl-6*H*-benzo[*f*]naphtho[2,3-*b*]phosphoindoles **2**

A solution of *n*-BuLi (1.63 M in hexane, 1.5 mL, 2.4 mmol, 2.4 equiv) was added dropwise to a solution of 3,3′-dibromo-2,2′-binaphthyl (**1**, 412 mg, 1 mmol) in dry THF (16 mL) at −78 °C under Ar atmosphere. After 10 min, to the reaction mixture, dichlorophenylphosphine (0.32 mL, 2.4 mmol, 2.4 equiv) was added, and the resulting mixture was stirred for 1 h. The reaction mixture was diluted with

CH_2Cl_2 (30 mL) and water (30 mL) at 0 °C. The phases were separated, and the aqueous layer was extracted with CH_2Cl_2 (30 mL × 3). The combined organic layer was washed with water (30 mL × 3), dried over anhydrous magnesium sulfate, filtered, and concentrated under reduced pressure. The residue was purified by column chromatography using *n*-hexane/CH_2Cl_2 4:1 as eluent to give **2** as colorless prisms (189 mg, 52%). mp 214-216 °C (CH_2Cl_2/*n*-hexane). ^{1}H NMR (400 MHz, $CDCl_3$) δ: 8.54 (s, 2H, Ar-H), 8.20 (d, J = 5.9 Hz, 2H, Ar-H), 7.98 (d, J = 7.8 Hz, 2H, Ar-H), 7.83 (d, J = 7.8 Hz, 2H, Ar-H), 7.54-7.46 (m, 4H, Ar-H), 7.36 (t, J = 7.8 Hz, 2H, Ar-H), 7.21 (d, J = 7.3 Hz, 3H, Ar-H). ^{13}C NMR (100 MHz, $CDCl_3$) δ: 141.1 (C, s), 140.6 (C, d, $J_{C,P}$ = 4.1 Hz), 138.3 (C, d, $J_{C,P}$ = 21.5 Hz), 133.8 (C), 133.6 (C, d, $J_{C,P}$ = 8.2 Hz), 132.6 (CH, d, $J_{C,P}$ = 19.9 Hz), 130.8 (CH, d, $J_{C,P}$ = 24.0 Hz), 129.2 (CH), 128.6 (CH, d, $J_{C,P}$ = 7.4 Hz), 128.4 (CH), 128.0 (CH), 126.6 (CH), 126.1 (CH), 120.2 (CH). ^{31}P{^{1}H} NMR (161 MHz, $CDCl_3$) δ: −13.27 (s). FTIR (KBr): 3053, 1431, 874, 741 cm^{-1}. LRMS (EI) *m/z*: 360 ([M]$^+$). HRMS: *m/z* [M]$^+$ calcd for $C_{26}H_{17}P$: 360.1068. Found: 360.1052.

6-Phenyl-6*H*-benzo[*f*]naphtho[2,3-*b*]phosphoindole oxide (**3**) [1]

To a solution of 6-phenyl-6*H*-benzo[*f*]naphtho[2,3-*b*]phosphoindole (**2**, 180 mg, 0.5 mmol) in dry CH_2Cl_2 (5 mL), hydrogen peroxide (30% solution in water, 0.6 mL, 5.9 mmol, 12 equiv) was added, and this was stirred at 0 °C under air. After 30 min, the mixture was stirred at room temperature for 30 min. The reaction mixture was diluted with CH_2Cl_2 (10 mL) and water (15 mL). The aqueous phase was extracted with CH_2Cl_2 (10 mL × 2). The combined extracts were washed with water (10 mL × 2) and brine (10 mL × 2), dried over anhydrous magnesium sulfate, filtered, and concentrated under reduced pressure. The residue was purified by recrystallization to give **3** as colorless plates (173 mg, 92%). mp > 300 °C (CH_2Cl_2/*n*-hexane), (Lit. 325 °C [1]). ^{1}H NMR (400 MHz, $CDCl_3$) δ: 8.40 (d, J = 2.4 Hz, 2H, Ar-H), 8.27 (d, J = 11.2 Hz, 2H, Ar-H), 7.93 (d, J = 8.3 Hz, 2H, Ar-H), 7.81 (d, J = 8.3 Hz, 2H, Ar-H), 7.71 (dd, J = 7.8, 13.1 Hz, 2H, Ar-H), 7.54 (t, J = 7.8 Hz, 2H, Ar-H), 7.47 (t, J = 7.8 Hz, 3H, Ar-H), 7.38 (td, J = 2.4, 7.3 Hz, 2H, Ar-H). ^{13}C NMR (100 MHz, $CDCl_3$) δ: 137.5 (C, d, $J_{C,P}$ =

20.7 Hz), 136.0 (C, d, $J_{C,P}$ = 1.7 Hz), 133.6 (C, d, $J_{C,P}$ = 12.4 Hz), 132.1 (C, d, $J_{C,P}$ = 105.9 Hz), 132.0 (CH, d, $J_{C,P}$ = 2.5 Hz), 131.7 (CH, d, $J_{C,P}$ = 9.9 Hz), 131.1 (CH, d, $J_{C,P}$ = 10.7 Hz), 131.0 (C), 129.2 (CH), 128.7 (CH), 128.6 (CH), 128.5 (CH, d, $J_{C,P}$ = 2.5 Hz), 127.0 (CH), 120.3 (CH, d, $J_{C,P}$ = 9.1 Hz). $^{31}P\{^1H\}$ NMR (161 MHz, $CDCl_3$) *δ*: 32.29 (s). FTIR (KBr): 3032, 1194, 748, 731, 542 cm^{-1}. LRMS (EI) *m/z*: 376 ($[M]^+$). HRMS: *m/z* $[M]^+$ calcd for $C_{26}H_{17}P$: 376.1017. Found: 376.1019.

6-Phenyl-6*H*-benzo[*f*]naphtho[2,3-*b*]phosphoindole sulfide (**4**)

To a solution of 6-phenyl-6*H*-benzo[*f*]naphtho[2,3-*b*]phosphoindole (**2**, 125 mg, 0.35 mmol) in dry toluene (2 mL), element sulfur (55 mg, 1.7 mmol, 5 equiv) was added and stirred at 60 °C under air. After 1.5 h, the reaction mixture was diluted with CH_2Cl_2 (10 mL) and water (10 mL). The aqueous phase was extracted with CH_2Cl_2 (10 mL × 2). The combined extracts were washed with water (10 mL × 2), dried over anhydrous magnesium sulfate, filtered, and concentrated under reduced pressure. The residue was purified by column chromatography using *n*-hexane/CH_2Cl_2 1:1 as eluent to give **4** as pale yellow plates (119 mg, 88%). mp 298-300 °C (CH_2Cl_2/*n*-hexane). ^1H NMR (400 MHz, $CDCl_3$) *δ*: 8.43 (d, *J* = 3.2 Hz, 2H, Ar-H), 8.27 (d, *J* = 12.8 Hz, 2H, Ar-H), 7.95 (d, *J* = 7.8 Hz, 2H, Ar-H), 7.81 (d, *J* = 8.2 Hz, 2H, Ar-H), 7.76 (ddd, *J* = 1.4, 8.0, 14.6 Hz, 2H, Ar-H), 7.56 (td, *J* = 0.9, 8.2 Hz, 2H, Ar-H), 7.47 (td, *J* = 0.9, 8.2 Hz, 2H, Ar-H), 7.41 (td, *J* = 2.3, 7.3 Hz, 1H, Ar-H), 7.33 (td, *J* = 3.2, 7.8 Hz, 2H, Ar-H). ^{13}C NMR (100 MHz, $CDCl_3$) *δ*: 137.3 (C, d, $J_{C,P}$ = 18.3 Hz), 135.6 (C), 134.1 (C, d, $J_{C,P}$ = 90.5 Hz), 133.8 (C, d, $J_{C,P}$ = 12.5 Hz), 132.7 (C, d, $J_{C,P}$ = 81.7 Hz), 131.7 (CH, d, $J_{C,P}$ = 3.0 Hz), 131.2 (CH, d, $J_{C,P}$ = 10.6 Hz), 131.0 (CH, d, $J_{C,P}$ = 11.6 Hz), 129.0 (CH), 128.52 (CH), 128.51 (CH, d, $J_{C,P}$ = 13.5 Hz), 128.4 (CH), 127.0 (CH), 120.5 (CH, d, $J_{C,P}$ = 9.6 Hz). $^{31}P\{^1H\}$ NMR (161 MHz, $CDCl_3$) *δ*: 39.27 (s). FTIR (KBr): 3055, 1508, 1497, 1437, 883, 744 cm^{-1}. LRMS (EI) *m/z*: 393.05 ($[M+H]^+$). HRMS: *m/z* $[M]^+$ calcd for $C_{26}H_{17}PS$: 392.0789. Found: 392.0792.

6-Methyl-6-phenyl-6*H*-benzo[*f*]naphtho[2,3-*b*]phosphoindorium trifluoromethansulfonate (**5**)

To a solution of 6-phenyl-6*H*-benzo[*f*]naphtho[2,3-*b*]phosphoindole (**2**, 126 mg, 0.35 mmol) in dry CH_2Cl_2 (10 mL), methyl trifluoromethanesulfonate (47 μL, 0.43 mmol, 1.2 equiv) was added and stirred at room temperature. After 2 h, the reaction mixture was concentrated under reduced pressure. The residue was washed with *n*-hexane and purified by recrystallization from a toluene/methanol mixture to give **5** as colorless plates (149 mg, 81%). mp 295-297 °C (toluene/methanol). ^{1}H NMR (400 MHz, CD_3OD) δ: 8.90 (d, J = 2.7 Hz, 2H, Ar-H), 8.81 (d, J = 12.4 Hz, 2H, Ar-H), 8.18 (d, J = 8.3 Hz, 2H, Ar-H), 8.07 (d, J = 7.8 Hz, 2H, Ar-H), 7.94 (dd, J = 7.3, 14.2 Hz, 2H, Ar-H), 7.83-7.78 (m, 3H, Ar-H), 7.73-7.68 (m, 4H, Ar-H), 2.99 (s, 3H, Me). ^{13}C NMR (100 MHz, $CDCl_3$) δ: 139.7 (C, d, $J_{C,P}$ = 18.3 Hz), 138.4 (C), 136.4 (CH, d, $J_{C,P}$ = 2.9 Hz), 136.0 (CH, d, $J_{C,P}$ = 10.6 Hz), 135.1 (C, d, $J_{C,P}$ = 13.4 Hz), 133.3 (CH, d, $J_{C,P}$ = 11.5 Hz), 131.7 (CH), 131.6 (CH, d, $J_{C,P}$ = 13.5 Hz), 130.5 (CH), 130.2 (CH), 129.5 (CH), 123.9 (CH, d, $J_{C,P}$ = 8.7 Hz), 121.5 (C, d, $J_{C,P}$ = 87.1 Hz), 121.4 (C, d, $J_{C,P}$ = 94.4 Hz), 8.2 (CH_3, d, $J_{C,P}$ = 53.0 Hz). ^{31}P{^{1}H} NMR (161 MHz, $CDCl_3$) δ: 22.79 (s). FTIR (KBr): 3061, 2914, 1282, 1253, 1030, 637 cm^{-1}. LRMS (EI) *m/z*: 375.15 ([M-OTf]$^{+}$). HRMS: *m/z* [M]$^{+}$ calcd for $C_{27}H_{20}P$: 375.1303. Found: 375.1300.

6-Phenyl-6*H*-benzo[*f*]naphtho[2,3-*b*]phosphoindole borane complex **6**

To a solution of 6-phenyl-6*H*-benzo[*f*]naphtho[2,3-*b*]phosphoindole (**2**, 126 mg, 0.35 mmol) in dry THF (5 mL), borane THF complex (1.0 M in THF solution, 0.7 mL, 0.7 mmol, 2 equiv) was added at 0 °C and stirred. After 15 min, the reaction mixture was stirred at room temperature for 3 h. Then, the reaction mixture was concentrated under reduced pressure. The residue was concentrated under reduced pressure and purified by column chromatography using *n*-hexane/CH_2Cl_2 1:1 as eluent to give **6** as a colorless plates (120 mg, 91%). mp 294-296 °C (CH_2Cl_2/*n*-hexane). ^{1}H NMR (400 MHz, $CDCl_3$) δ: 8.49 (s, 2H, Ar-H), 8.25 (d, J = 9.7 Hz, 2H, Ar-H), 7.96 (d, J = 7.8 Hz, 2H, Ar-H), 7.83 (d, J = 7.8 Hz, 2H, Ar-H), 7.65-7.55 (m, 4H, Ar-H), 7.50 (td, J = 1.3, 7.3 Hz, 2H, Ar-H), 7.41 (td, J = 2.3, 7.3 Hz,

1H, Ar-H), 7.32 (td, J = 2.3, 7.3 Hz, 2H, Ar-H). ^{13}C NMR (100 MHz, $CDCl_3$) δ: 139.3 (C, d, $J_{C,P}$ = 9.6 Hz), 135.2 (C), 133.6 (C, d, $J_{C,P}$ = 11.5 Hz), 130.9(C), 132.2 (CH, d, $J_{C,P}$ = 10.6 Hz), 132.0 (CH, d, $J_{C,P}$ = 12.5 Hz), 131.6 (CH, d, $J_{C,P}$ = 3.9 Hz), 129.8 (C, d, $J_{C,P}$ = 51.0 Hz), 128.9 (CH, d, $J_{C,P}$ = 10.5 Hz), 128.6 (CH), 128.5 (CH), 128.2 (CH), 126.9 (CH), 120.8 (CH, d, $J_{C,P}$ = 5.8 Hz). $^{31}P\{^1H\}$ NMR (161 MHz, $CDCl_3$) δ: 22.53 (s). FTIR (KBr): 3055, 2376, 1055, 881, 741 cm^{-1}. Anal. Calc. for $C_{26}H_{20}BP$: C, 83.45; H, 5.39. Found: C, 83.47; H, 1.47.

6-Phenyl-6*H*-benzo[*f*]naphtho[2,3-*b*]phosphoindole–gold(I) chloride complex **7**

To a solution of chloro(dimethyl sulfide)gold(I) (87 mg, 0.3 mmol) in dry CH_2Cl_2 (6 mL), 6-phenyl-6*H*-benzo[*f*]naphtho[2,3-*b*]phosphoindole (**2**, 127 mg, 0.35 mmol, 1.1 equiv) was added and stirred at room temperature. After 3 h, the reaction mixture was concentrated under reduced pressure. The residue was washed with *n*-hexane and acetone and purified by recrystallization to give **7** as colorless powder (81 mg, 39%). mp 283-286 °C ($CHCl_3$). ^1H NMR (400 MHz, $CDCl_3$) δ: 8.48 (s, 2H, Ar-H), 8.22 (d, J = 11.9 Hz, 2H, Ar-H), 7.98 (d, J = 8.3 Hz, 2H, Ar-H), 7.82 (d, J = 8.3 Hz, 2H, Ar-H), 7.62-7.50 (m, 6H, Ar-H), 7.44 (t, J = 7.3 Hz, 1H, Ar-H), 7.34 (t, J = 7.3 Hz, 2H, Ar-H). ^{13}C NMR (100 MHz, $CDCl_3$) δ: 138.8 (C, d, $J_{C,P}$ = 9.5 Hz), 135.2 (C), 133.7 (CH, d, $J_{C,P}$ = 15.4 Hz), 133.4 (C, d, $J_{C,P}$ = 13.5 Hz), 133.1 (CH, d, $J_{C,P}$ = 14.4 Hz), 132.4 (CH), 130.4 (C, d, $J_{C,P}$ = 67.4 Hz), 129.4 (CH, d, $J_{C,P}$ = 12.5 Hz), 128.72 (CH), 128.68 (CH), 128.5 (CH), 127.3 (CH), 121.0 (CH, d, $J_{C,P}$ = 6.7 Hz). $^{31}P\{^1H\}$ NMR (161 MHz, $CDCl_3$) δ: 23.46 (s). FTIR (KBr): 3050, 1497, 1435, 883, 742 cm^{-1}. LRMS (EI) *m/z*: 598.15 ($[M+Li]^+$). HRMS: *m/z* $[M]^+$ calcd for $C_{26}H_{17}AuClP$: 592.0422. Found: 592.0422.

3. Single crystal X-ray diffraction experiment

Crystals were immersed in Paraton-N oil and placed in the N_2 cold stream at 100 K. The diffraction experiment was performed on a Bruker D8 VENTURE system (Cu Kα: λ = 1.54178 Å). Absorption correction was performed by an empirical method implemented in SADABS [2]. Structure solution and refinement were performed by using SHELXT-2014/5 [3] and SHELXL-2014/7 [4].

Crystal data and structure refinement for 2

The colorless plate crystal (0.200 × 0.050 × 0.020 mm^3) was obtained from dichloromethane/hexane. $C_{26}H_{17}P$, Mr = 451.14; monoclinic, space group $P2_1/n$, Z = 4, D_{calc} = 1.341 g·cm^{-3}, a = 12.2188(7), b = 8.1128(5), c = 18.0118(10) Å, β = 91.876(2)°, V = 1784.53(18) $Å^3$, 23358 measured and 3789 independent [$I > 2\sigma(I)$] reflections, 244 parameters, final R_1 = 0.0347, wR_2 = 0.0919, S = 1.049 [$I > 2\sigma(I)$]. CCDC 2051842.

All nonhydrogen atoms were refined anisotropically. The hydrogen atoms were refined isotropically on the calculated positions using a riding model (AFIX 43) with U_{iso} values constrained to 1.2 U_{eq} of the parent atoms.

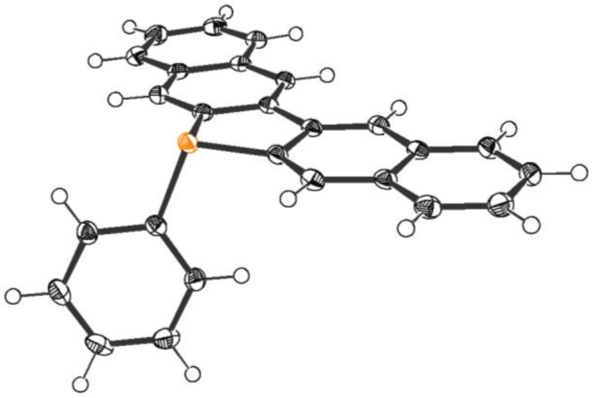

Figure S1: Ortep drawing of **2** (50% probability).

Crystal data and structure refinement for *N*-phenyldibenzo[*b*,*h*]carbazole

The colorless plate crystal (0.120 × 0.100 × 0.070 mm^3) wasobtained from dichloromethane/hexane. $C_{26}H_{17}N$, *M*r = 343.40; monoclinic, space group $P2_1/n$, $Z = 8$, $D_{calc} = 1.335$ g·cm^{-3}, $a = 9.7796(4)$, $b = 19.3772(9)$, $c = 18.4168(8)$ Å, $\beta = 101.827(2)°$, $V = 3415.9(3)$ Å^3, 45118 measured and 6725 independent [$I > 2\sigma(I)$] reflections, 487parameters, final $R_1 = 0.0356$, $wR_2 = 0.0920$, $S = 1.022$ [$I > 2\sigma(I)$]. CCDC 2051843

All nonhydrogen atoms were refined anisotropically. The hydrogen atoms were refined isotropically on the calculated positions using a riding model (AFIX 43) with U_{iso} values constrained to 1.2 U_{eq} of the parent atoms.

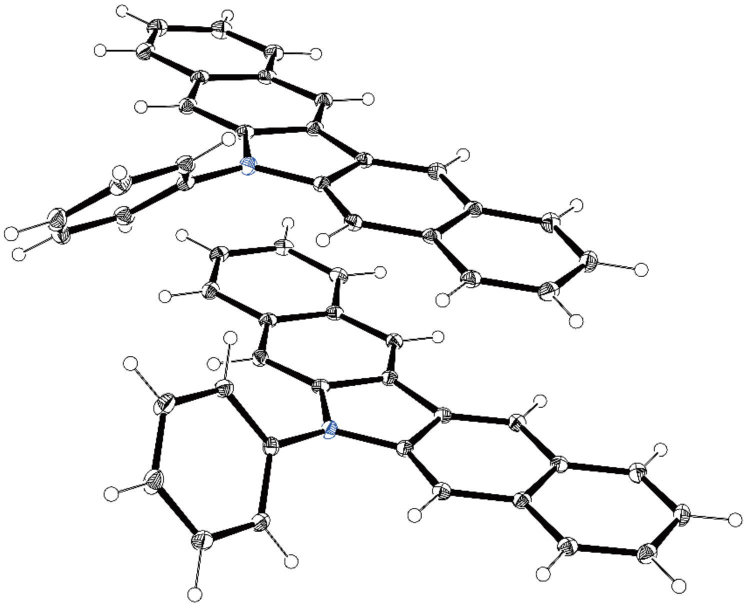

Figure S2: Ortep drawing of *N*-phenyldibenzo[*b*,*h*]carbazole (50% probability).

4. Fluorescence spectra of all product

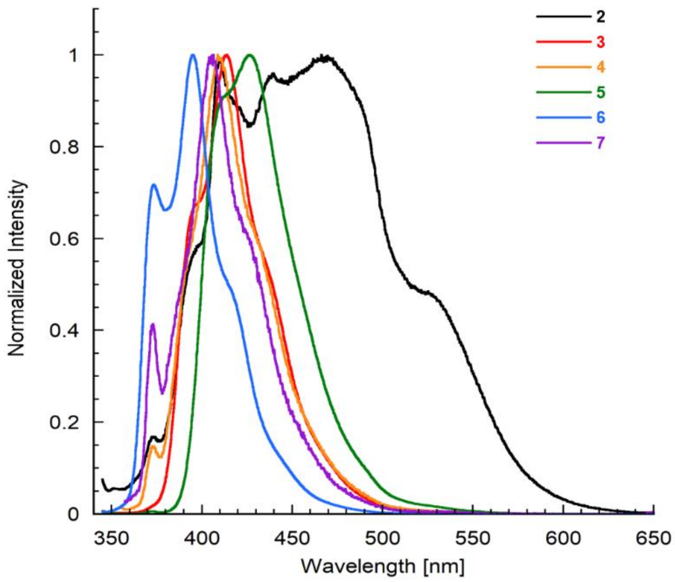

Figure S3: Normalized fluorescence spectra in $CHCl_3$ (excited at 335 nm).

5. Cyclic voltammograms of all products

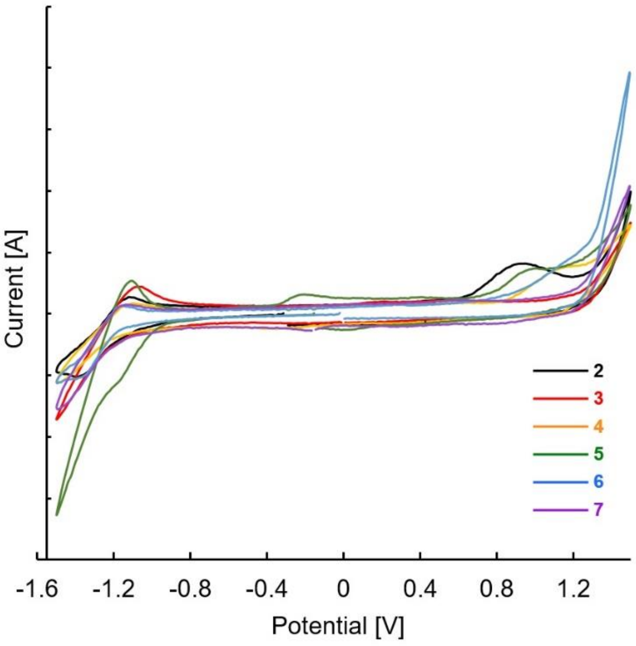

Figure S4: Cyclic voltammograms in DCB.

6. Computational details

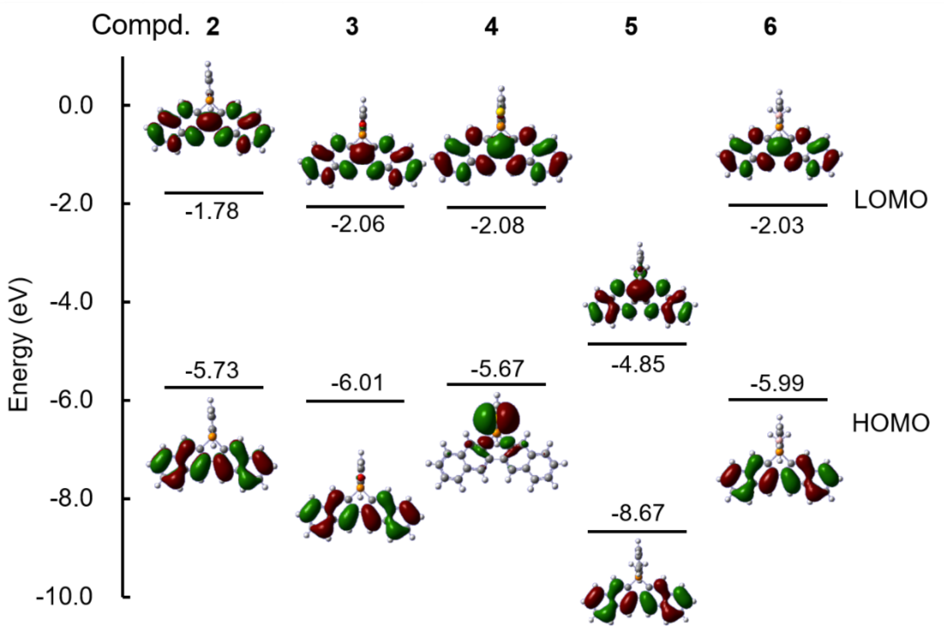

Figure S5: The spatial plots and energy levels of the HOMO and LUMO of selected compounds. The calculations were performed at the level of B3LYP/LanL2DZ.

Table S1: Excitation energies and oscillator strengths calculated by the TDDFT method.

compound	state	wavelength (nm)	oscillator strength	excitation	weight
**2**	1	346.19	0.0095	HUMO−1 >> LUMO	88%
	2	339.15	0.1999	HOMO >> LUMO	93%
	3	320.47	0.0061	HOMO−2 >> LUMO	50%
**3**	1	344.05	0.0573	HOMO >> LUMO	78%
	2	331.45	0.0496	HOMO−2 >> LUMO	43%
				HOMO >> LUMO+1	35%
	3	318.67	0.0027	HUMO−1 >> LUMO	73%
**4**	1	410.40	0.0031	HOMO >> LUMO	96%
	2	406.67	0.0009	HUMO−1 >> LUMO	97%
	3	370.11	0.0026	HOMO >> LUMO+1	93%
**5**	1	372.27	0.0073	HOMO >> LUMO	94%
	2	337.46	0.2071	HOMO >> LUMO+1	66%
	3	328.91	0.0006	HOMO >> LUMO+2	99%
**6**	1	340.50	0.1423	HOMO >> LUMO	90%
	2	323.80	0.0058	HUMO−1 >> LUMO	72%
	3	323.01	0.0530	HOMO >> LUMO+1	52%

All calculations were performed at the DFT level, by means of the B3LYP functional as implemented in Gaussian 16. [5] The LanL2DZ basis sets were used. The X-ray structure of **2** was used as initial structures for geometry optimization calculations.

Cartesian coordinates and energies of **2**

E(B3LYP) = -1007.24434090 A.U.

C	-1.31196	0.14151	-0.82171	H	3.41672	-3.82144	1.19775
C	-0.75174	-1.04601	-0.22337	C	5.22881	-2.77776	0.68449
C	-1.59821	-2.02864	0.27908	H	5.88375	-3.54915	1.08216
H	-1.19207	-2.93271	0.72865	C	5.79777	-1.60726	0.09529
C	-3.01639	-1.87241	0.21968	H	6.87886	-1.50086	0.05115
C	-3.90923	-2.86582	0.73456	C	4.97477	-0.61258	-0.41785
H	-3.49252	-3.76549	1.18317	H	5.40665	0.27931	-0.86739
C	-5.28518	-2.69068	0.66595	C	3.55154	-0.74032	-0.36612
H	-5.95419	-3.45162	1.06032	C	2.68104	0.26659	-0.89446
C	-5.83266	-1.50993	0.07679	H	3.12229	1.14982	-1.35232
H	-6.91167	-1.38545	0.02934	P	0.00777	1.30098	-1.50598
C	-4.99151	-0.52844	-0.43226	C	0.02263	2.69942	-0.21806
H	-5.40692	0.37116	-0.88195	C	0.15042	4.02101	-0.69675
C	-3.57077	-0.68015	-0.37625	C	-0.08398	2.47719	1.17241
C	-2.68187	0.3127	-0.90043	C	0.17577	5.108	0.20111
H	-3.10673	1.20366	-1.35873	H	0.22822	4.20146	-1.76698
C	1.30845	0.11846	-0.81928	C	-0.06043	3.56054	2.06878
C	0.7265	-1.05844	-0.22114	H	-0.18744	1.46458	1.55455
C	1.55471	-2.05451	0.28511	C	0.07047	4.87884	1.58504
H	1.13216	-2.95109	0.73458	H	0.27497	6.12207	-0.17934
C	2.97548	-1.92219	0.22985	H	-0.14328	3.37878	3.13791
C	3.8499	-2.92963	0.74901	H	0.08861	5.71539	2.27997

Cartesian coordinates and energies of **3**

E(B3LYP) = -1082.44015671 A.U.

C	1.32260	0.06677	0.62261	C	-5.26040	-2.90540	-0.61189
C	0.74251	-1.13659	0.08597	H	-5.92187	-3.69866	-0.95114
C	1.57903	-2.15499	-0.35249	C	-5.81973	-1.71124	-0.06391
H	1.16719	-3.07468	-0.76258	H	-6.89948	-1.60906	0.00812
C	3.00000	-2.02028	-0.26681	C	-4.98835	-0.68830	0.37525
C	3.88220	-3.05566	-0.71020	H	-5.41151	0.22178	0.79504
H	3.45775	-3.96707	-1.12619	C	-3.56773	-0.81293	0.28594
C	5.25975	-2.90632	-0.61186	C	-2.68819	0.22679	0.72924
H	5.92109	-3.69968	-0.95115	H	-3.11176	1.13591	1.15083
C	5.81928	-1.71230	-0.06378	C	0.00035	2.70479	-0.04251
H	6.89905	-1.61032	0.00829	C	0.00066	4.00131	0.50730
C	4.98808	-0.68924	0.37542	C	0.00030	2.52160	-1.43942
H	5.41139	0.22073	0.79529	C	0.00098	5.12056	-0.34688
C	3.56743	-0.81361	0.28607	H	0.00064	4.11149	1.58922
C	2.68807	0.22625	0.72941	C	0.00063	3.64264	-2.28939
H	3.11182	1.13527	1.15106	H	0.00003	1.52065	-1.86610
C	-1.32274	0.06704	0.62248	C	0.00097	4.94233	-1.74353
C	-0.74286	-1.13646	0.08594	H	0.00123	6.12303	0.07417
C	-1.57955	-2.15473	-0.35247	H	0.00060	3.50482	-3.36791
H	-1.16788	-3.07454	-0.76247	H	0.00122	5.80753	-2.40266
C	-3.00050	-2.01974	-0.26684	P	0.00001	1.27109	1.15758
C	-3.88287	-3.05500	-0.71018	O	-0.00009	1.82117	2.67247
H	-3.45858	-3.96652	-1.12609				

Cartesian coordinates and energies of **4**

E(B3LYP) = -1017.36608899 A.U.

C	1.32263	0.02299	0.51108	H	3.45810	-4.08981	-1.04083
C	0.74109	-1.20626	0.03709	C	5.25975	-2.99937	-0.59192
C	1.57899	-2.24436	-0.35090	H	5.92149	-3.80729	-0.89395
H	1.16666	-3.18450	-0.71111	C	5.81884	-1.77735	-0.10852
C	2.99953	-2.10258	-0.27983	H	6.89868	-1.66915	-0.04761
C	3.88223	-3.15718	-0.67457	C	4.98745	-0.73533	0.28282

H	5.41038	0.19572	0.65381
C	3.56652	-0.86767	0.20765
C	2.68772	0.19097	0.60433
H	3.10979	1.11855	0.98462
C	-1.32291	0.02351	0.51077
C	-0.74176	-1.20599	0.03697
C	-1.57999	-2.24384	-0.35097
H	-1.16796	-3.18419	-0.71097
C	-3.00049	-2.10154	-0.28008
C	-3.88352	-3.15587	-0.67479
H	-3.45968	-4.08870	-1.04089
C	-5.26099	-2.99756	-0.59231
H	-5.92299	-3.80528	-0.89430
C	-5.81970	-1.77528	-0.10912
H	-6.89950	-1.66669	-0.04833
C	-4.98798	-0.73351	0.28219
H	-5.41062	0.19774	0.65303
C	-3.56709	-0.86637	0.20719
C	-2.68795	0.19200	0.60386
H	-3.10971	1.11976	0.98404
C	0.00078	2.59830	-0.33519
C	0.00144	3.94684	0.06298
C	0.00075	2.25861	-1.70420
C	0.00210	4.96272	-0.91273
H	0.00138	4.18624	1.12388
C	0.00140	3.27631	-2.67483
H	0.00022	1.21681	-2.01665
C	0.00209	4.62969	-2.28032
H	0.00262	6.00539	-0.60472
H	0.00136	3.01607	-3.73048
H	0.00260	5.41495	-3.03275
P	0.00002	1.25528	0.97828
S	-0.00055	2.03774	2.94620

Cartesian coordinates and energies of **5**

E(B3LYP) = -1046.94604757 A.U.

C	1.33079	0.04276	0.50773
C	0.74013	-1.20800	0.09558
C	1.58399	-2.25100	-0.26059
H	1.17562	-3.20789	-0.57636
C	3.00509	-2.09313	-0.22507
C	3.88491	-3.15883	-0.58941
H	3.46317	-4.11272	-0.89653
C	5.26249	-2.98231	-0.55297
H	5.92390	-3.79800	-0.83117
C	5.82340	-1.73201	-0.15149
H	6.90285	-1.61293	-0.12969
C	4.99533	-0.67652	0.20859
H	5.42250	0.27569	0.51437
C	3.57520	-0.82909	0.18210
C	2.69785	0.23941	0.54977
H	3.12942	1.19213	0.84960
C	-1.33455	0.04195	0.50492
C	-0.74229	-1.20835	0.09378
C	-1.58472	-2.25177	-0.26450
H	-1.17511	-3.20844	-0.57933
C	-3.00597	-2.09465	-0.23235
C	-3.88435	-3.16074	-0.59903
H	-3.46137	-4.11436	-0.90529
C	-5.26210	-2.98491	-0.56601
H	-5.92242	-3.80085	-0.84601
C	-5.82458	-1.73492	-0.16576
H	-6.90413	-1.61633	-0.14671
C	-4.99791	-0.67910	0.19655
H	-5.42631	0.27289	0.50130
C	-3.57767	-0.83099	0.17364

C	-2.70172	0.23793	0.54356
H	-3.13464	1.19045	0.84214
C	0.00331	2.80080	-0.03990
C	-0.04558	4.05936	0.59560
C	0.05399	2.70763	-1.44861
C	-0.04379	5.22991	-0.18568
H	-0.08411	4.14704	1.67785
C	0.05519	3.88201	-2.21909
H	0.09316	1.73951	-1.94225
C	0.00621	5.14254	-1.58939
H	-0.08102	6.20084	0.29967
H	0.09429	3.81569	-3.30245
H	0.00748	6.04830	-2.18930
P	-0.00232	1.23814	0.95179
C	-0.00689	1.64092	2.77069
H	0.88238	2.22260	3.03092
H	-0.90900	2.20209	3.03175
H	0.00341	0.69870	3.32467

Cartesian coordinates and energies of **6**

E(B3LYP) = -1033.88327825 A.U.

C	1.31875	0.07644	0.63498
C	0.74064	-1.13180	0.10231
C	1.57887	-2.14960	-0.33677
H	1.16637	-3.07256	-0.73890
C	2.99907	-2.00869	-0.26558
C	3.88248	-3.04179	-0.71302
H	3.45847	-3.95764	-1.11965
C	5.25991	-2.88444	-0.63041
H	5.92210	-3.67584	-0.97258
C	5.81831	-1.68411	-0.09431
H	6.89820	-1.57565	-0.03409
C	4.98632	-0.66347	0.34860
H	5.40915	0.25079	0.75945
C	3.56519	-0.79594	0.27511
C	2.68587	0.24050	0.72670
H	3.11331	1.14959	1.14364
C	-1.31874	0.07646	0.63493
C	-0.74065	-1.13179	0.10229
C	-1.57888	-2.14958	-0.33680
H	-1.16640	-3.07255	-0.73891
C	-2.99908	-2.00864	-0.26564
C	-3.88251	-3.04172	-0.71310
H	-3.45851	-3.95758	-1.11972
C	-5.25994	-2.88434	-0.63051
H	-5.92213	-3.67573	-0.97269
C	-5.81832	-1.68400	-0.09443
H	-6.89821	-1.57552	-0.03422
C	-4.98632	-0.66338	0.34850
H	-5.40913	0.25090	0.75934
C	-3.56519	-0.79587	0.27503
C	-2.68586	0.24055	0.72663
H	-3.11328	1.14965	1.14358
C	0.00005	2.67554	-0.07126
C	0.00019	4.00360	0.40174
C	0.00000	2.42170	-1.45955
C	0.00026	5.07472	-0.51348
H	0.00019	4.19494	1.47110
C	0.00009	3.49329	-2.36958
H	-0.00012	1.39973	-1.83143
C	0.00021	4.82190	-1.89762
H	0.00035	6.09773	-0.14540
H	0.00004	3.29452	-3.43854
H	0.00027	5.64955	-2.60312
P	0.00000	1.27837	1.18319
H	1.01196	2.55823	3.19734
H	-1.01362	2.55763	3.19652

H	-0.00047	0.89273	3.75319	B	-0.00059	1.90165	3.09371

---

7. Refereneces

1. Baba, K.; Tobisu, M.; Chatani, N. Chatani, *Org. Lett.*, **2014**, *17*, 70–73.
2. Sheldrick, G. M. (1996). *SADABS*. University of Göttingen, Germany.
3. Sheldrick, G. M. *Acta. Cryst.* **2015**, *A71*, 3-8.
4. Sheldrick, G. M. *Acta Cryst.* **2015**, *C71*, 3–8.
5. Gaussian 16, Revision C.01, Frisch, M. J.; Trucks, G. W.; Schlegel, H. B.; Scuseria, G. E.; Robb, M. A.; Cheeseman, J. R.; Scalmani, G.; Barone, V.; Petersson, G. A.; Nakatsuji, H.; Li, X.; Caricato, M.; Marenich, A. V.; Bloino, J.; Janesko, B. G.; Gomperts, R.; Mennucci, B.; Hratchian, H. P.; Ortiz, J. V.; Izmaylov, A. F.; Sonnenberg, J. L.; Williams-Young, D.; Ding, F.; Lipparini, F.; Egidi, F.; Goings, J.; Peng, B.; Petrone, A.; Henderson, T.; Ranasinghe, D.; Zakrzewski, V. G.; Gao, J.; Rega, N.; Zheng, G.; Liang, W.; Hada, M.; Ehara, M.; Toyota, K.; Fukuda, R.; Hasegawa, J.; Ishida, M.; Nakajima, T.; Honda, Y.; Kitao, O.; Nakai, H.; Vreven, T.; Throssell, K.; Montgomery, J. A., Jr.; Peralta, J. E.; Ogliaro, F.; Bearpark, M. J.; Heyd, J. J.; Brothers, E. N.; Kudin, K. N.; Staroverov, V. N.; Keith, T. A.; Kobayashi, R.; Normand, J.; Raghavachari, K.; Rendell, A. P.; Burant, J. C.; Iyengar, S. S.; Tomasi, J.; Cossi, M.; Millam, J. M.; Klene, M.; Adamo, C.; Cammi, R.; Ochterski, J. W.; Martin, R. L.; Morokuma, K.; Farkas, O.; Foresman, J. B.; Fox, D. J. Gaussian, Inc., Wallingford CT, 2016.

8. NMR spectra of products

^{1}H NMR spectrum of **2**

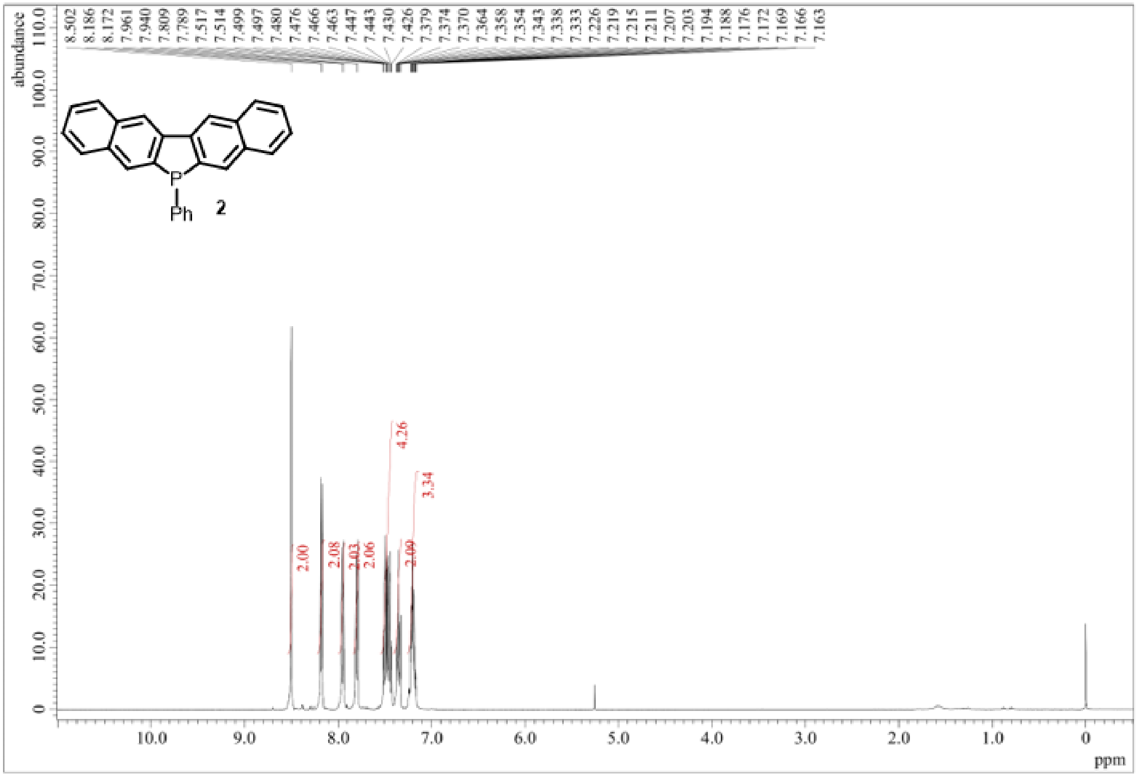

^{13}C NMR spectrum of **2**

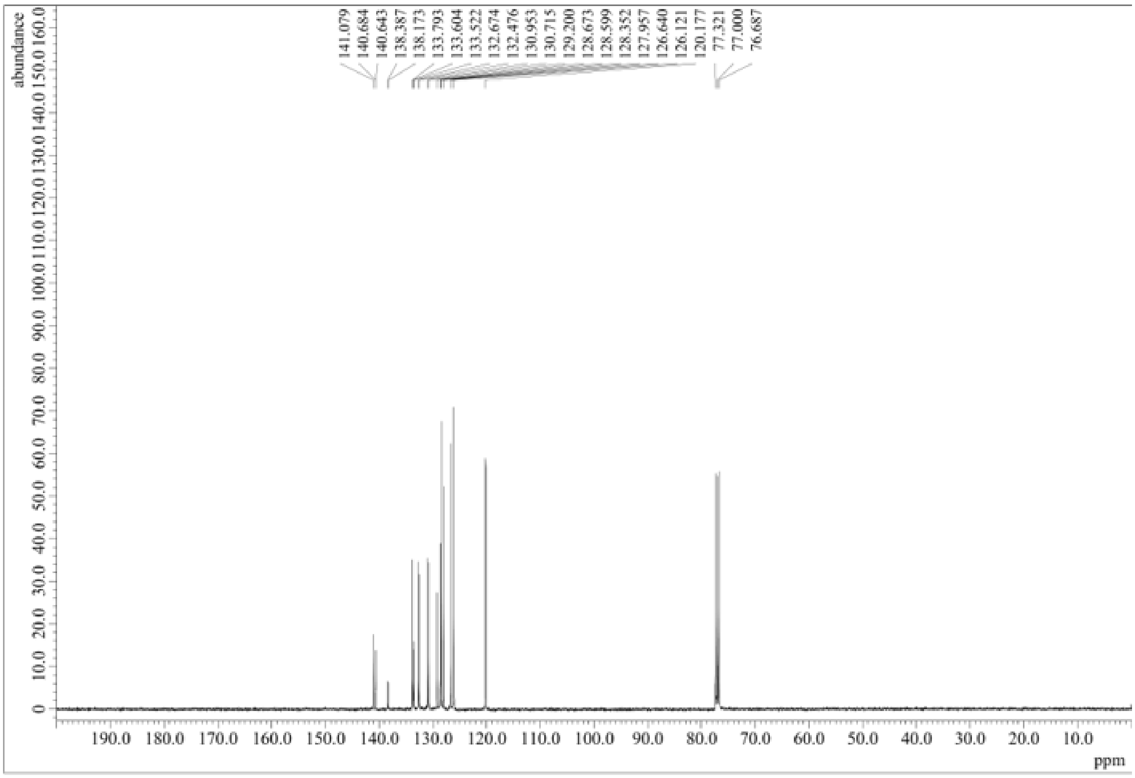

^{1}H NMR spectrum of **3**

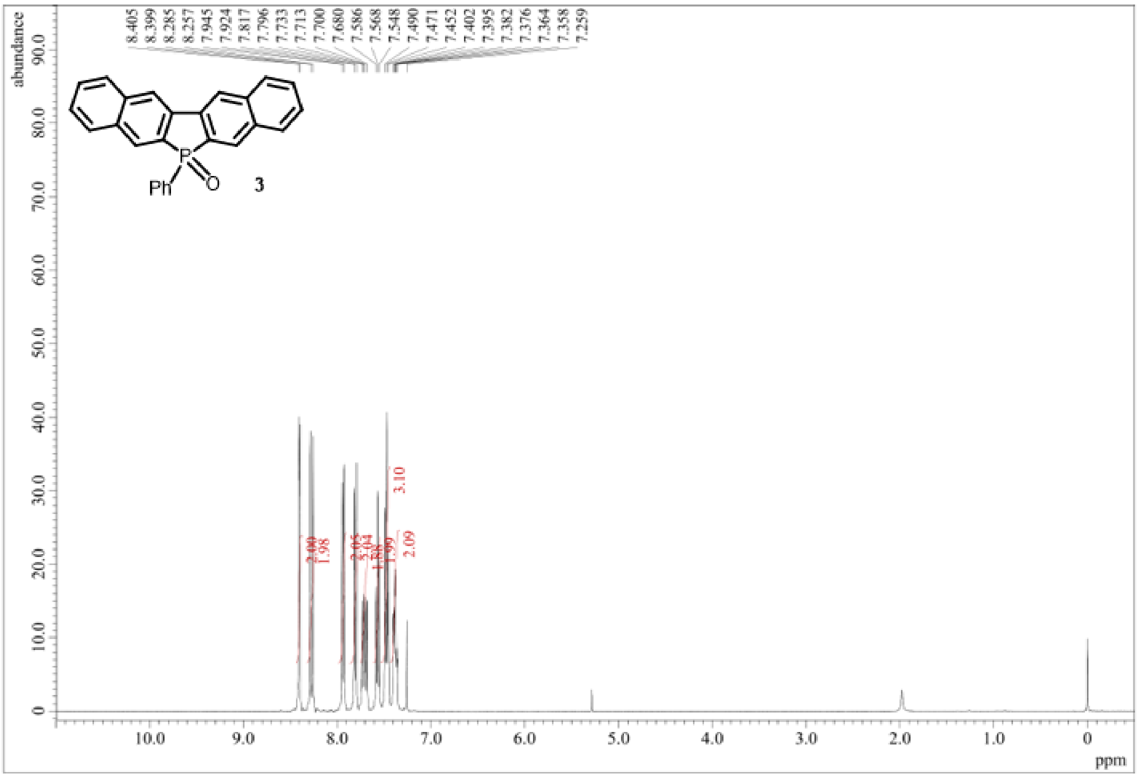

^{13}C NMR spectrum of **3**

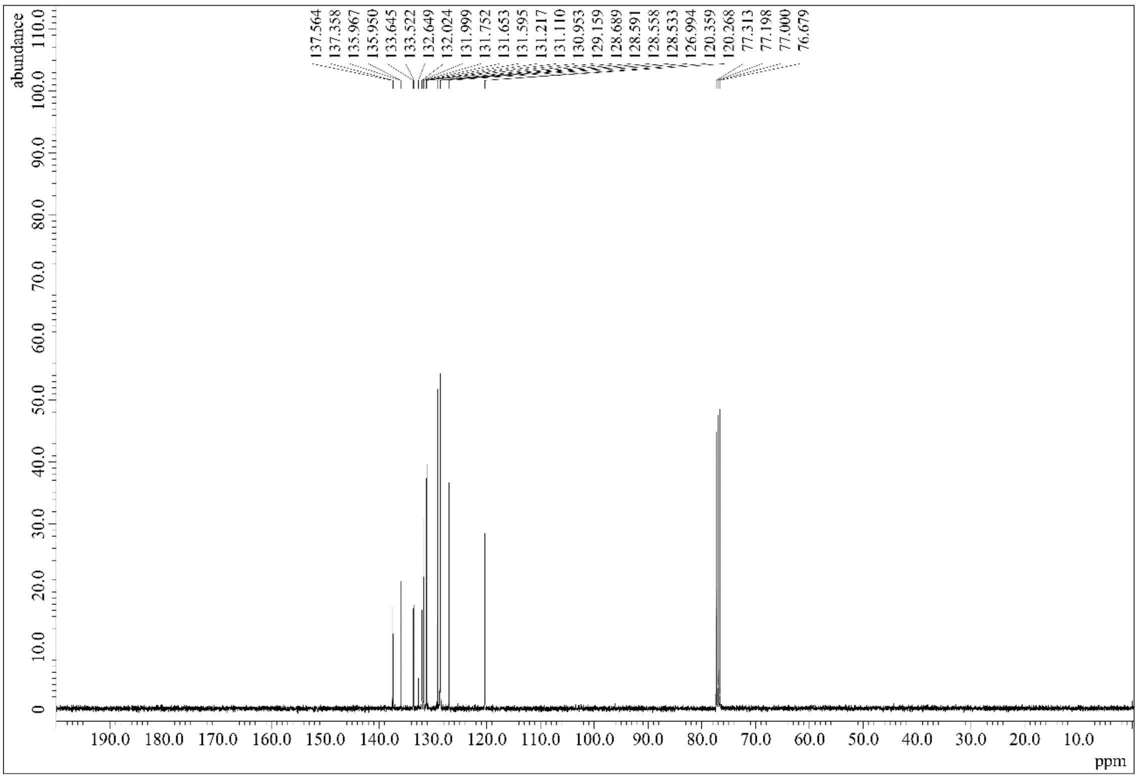

^{1}H NMR spectrum of **4**

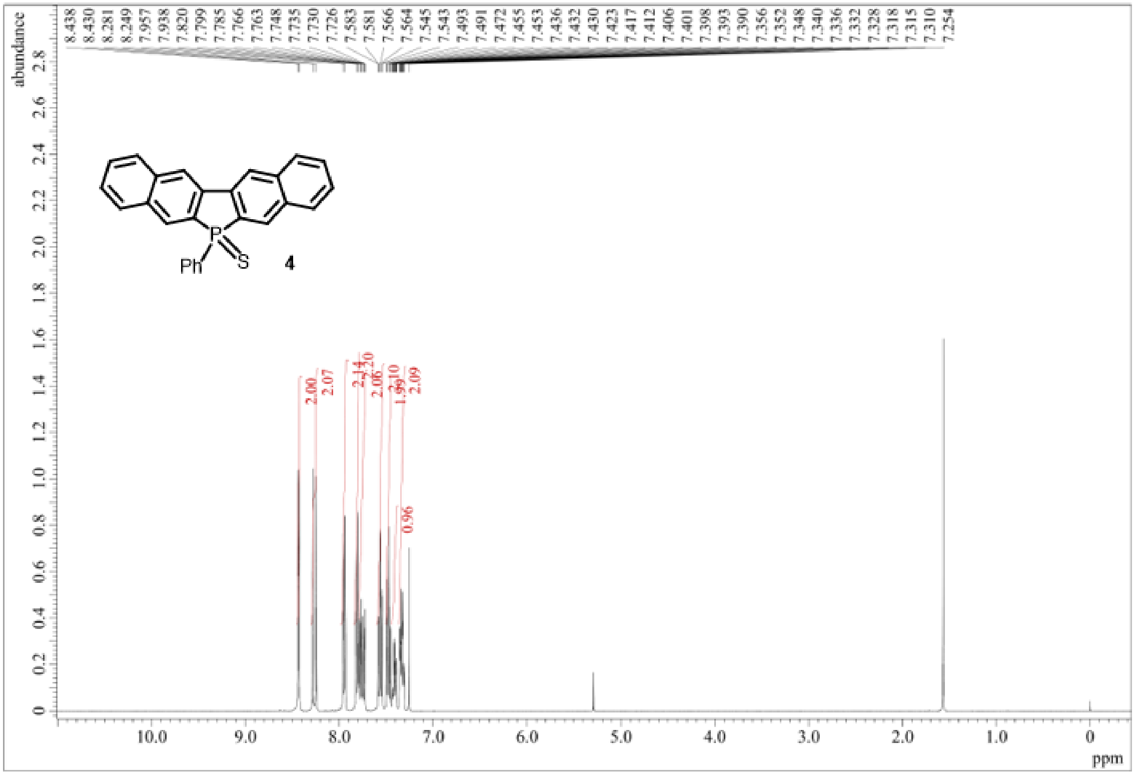

^{13}C NMR spectrum of **4**

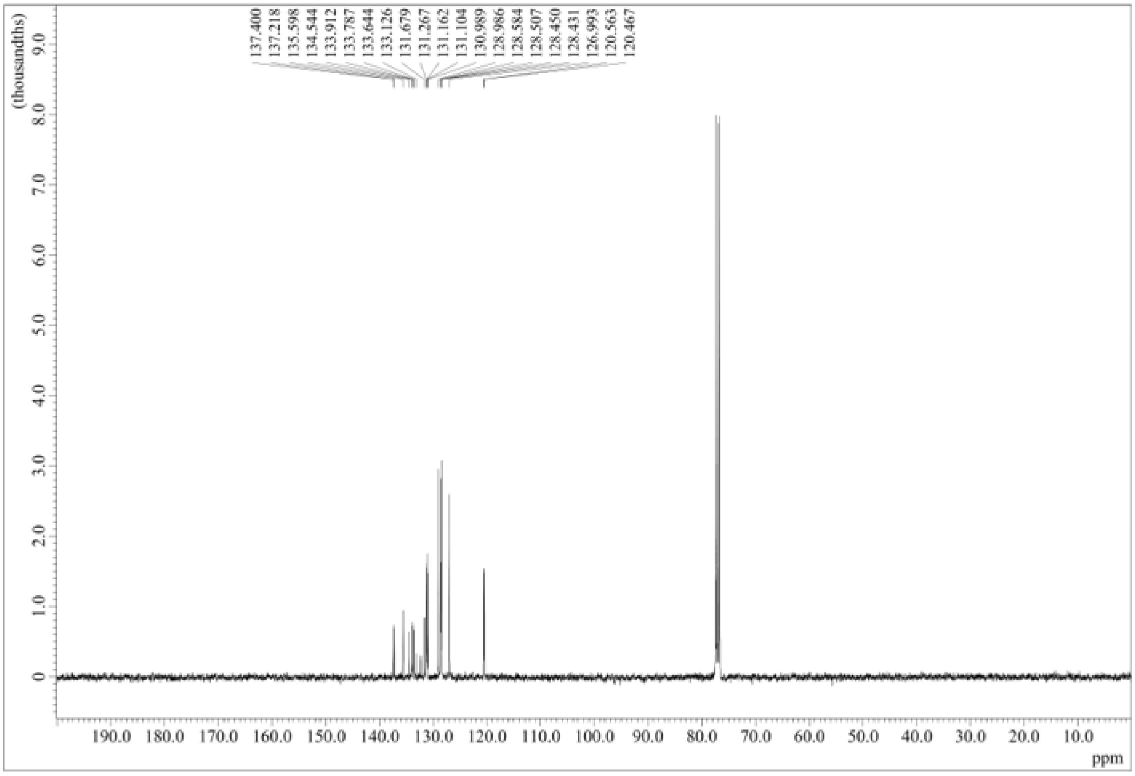

^{1}H NMR spectrum of **5**

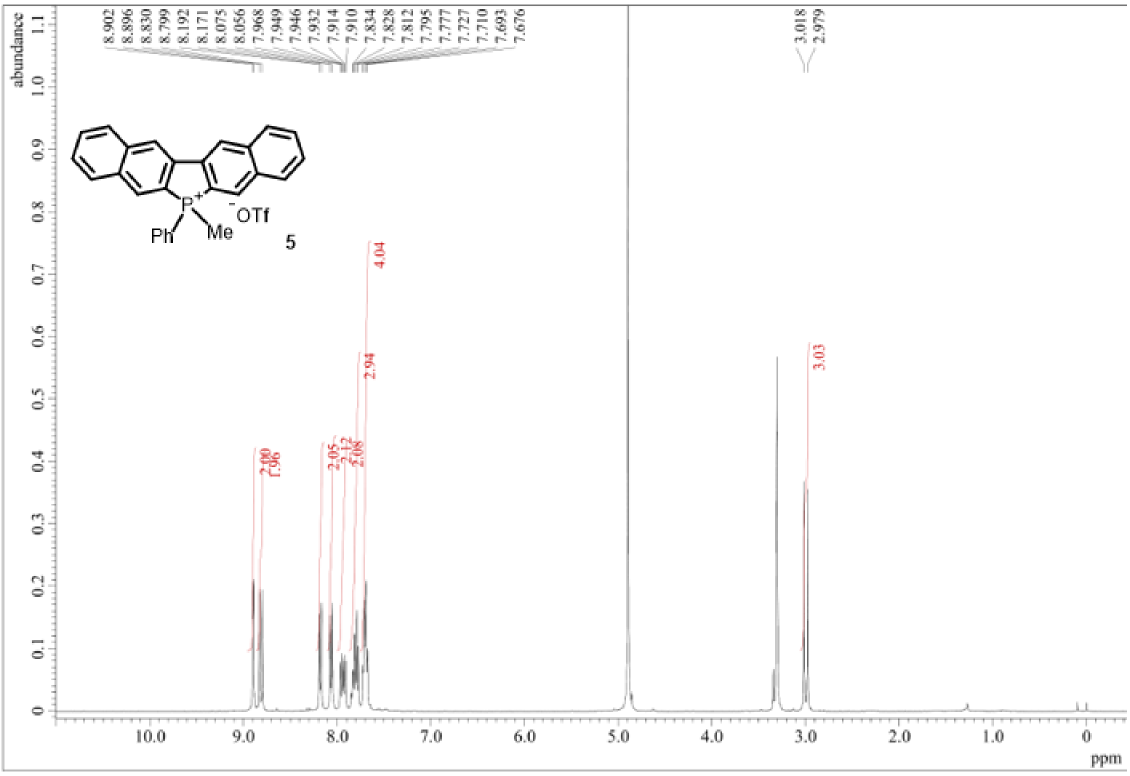

^{13}C NMR spectrum of **5**

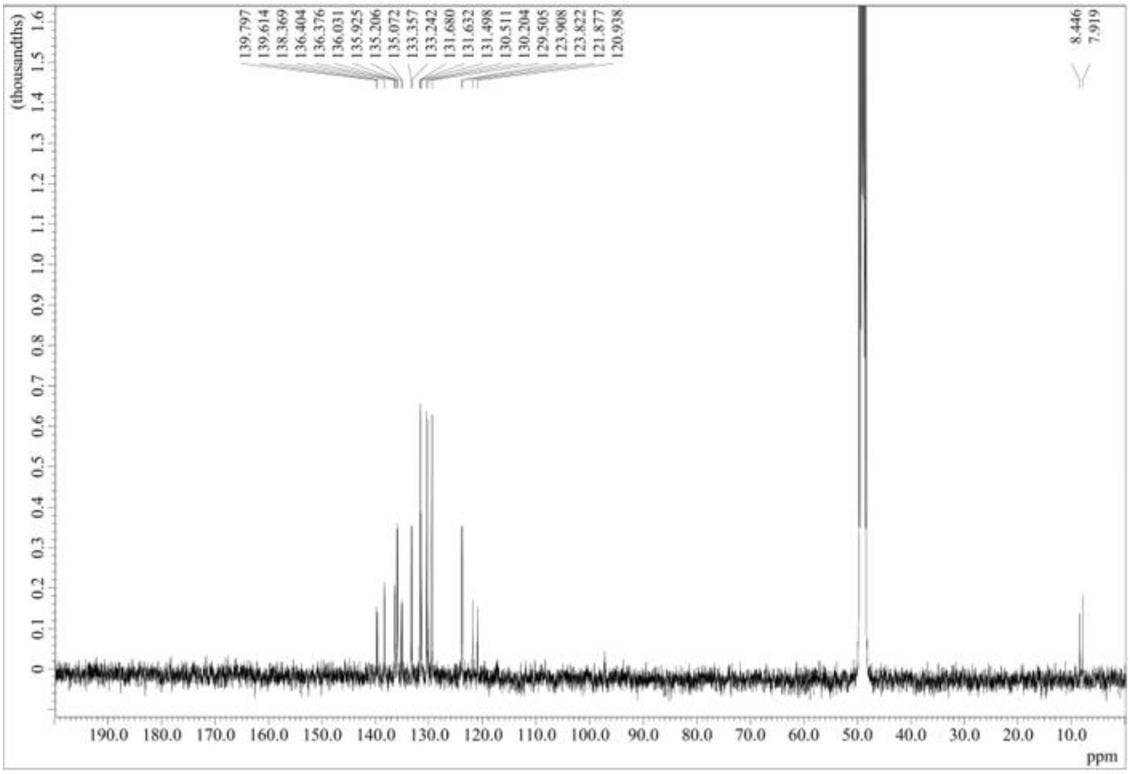

^{1}H NMR spectrum of **6**

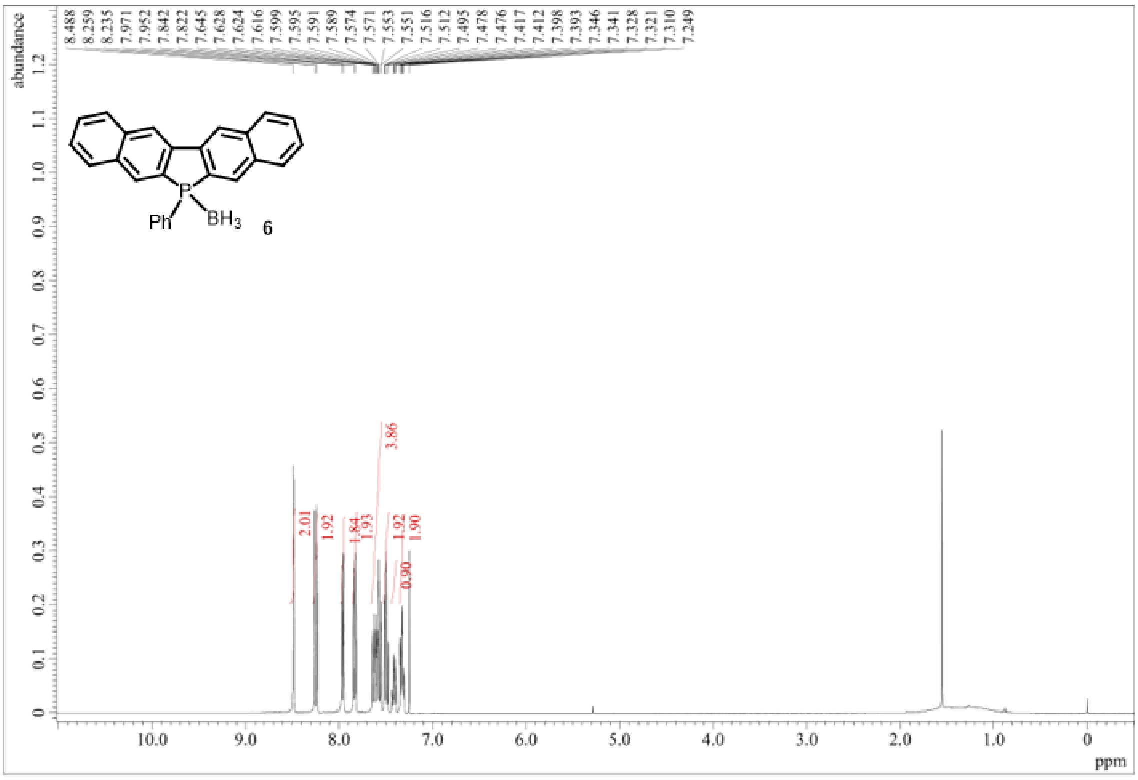

^{13}C NMR spectrum of **6**

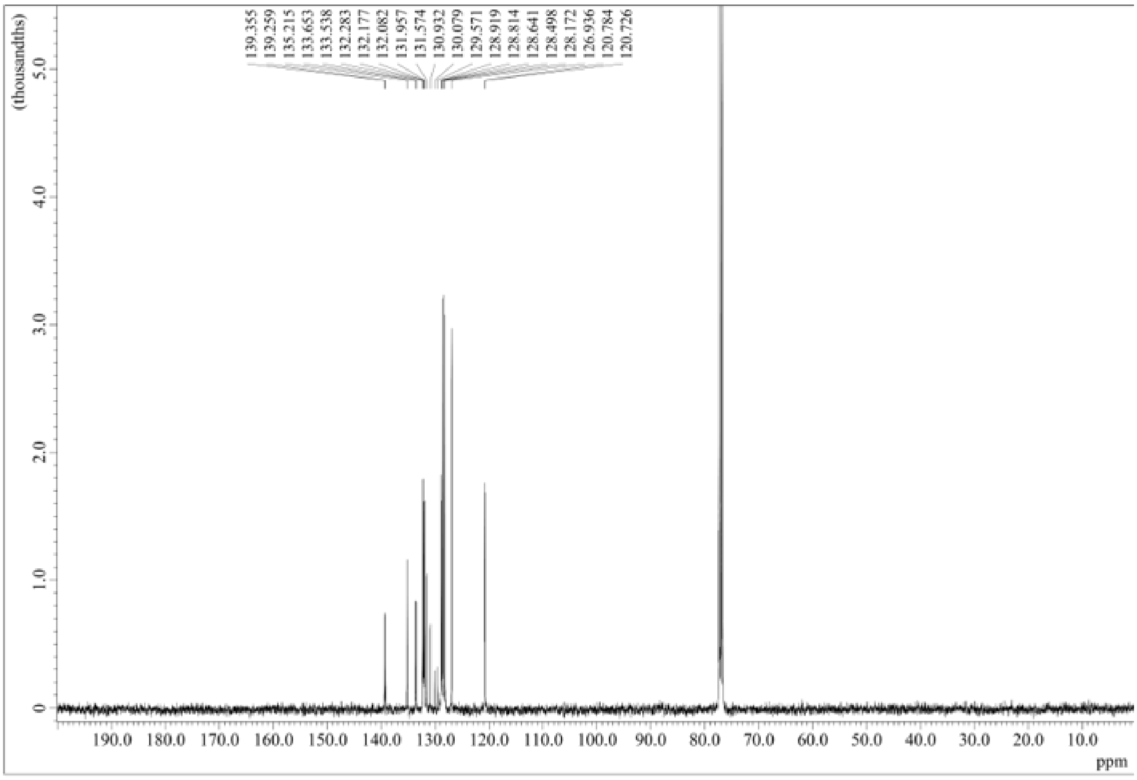

^{1}H NMR spectrum of **7**

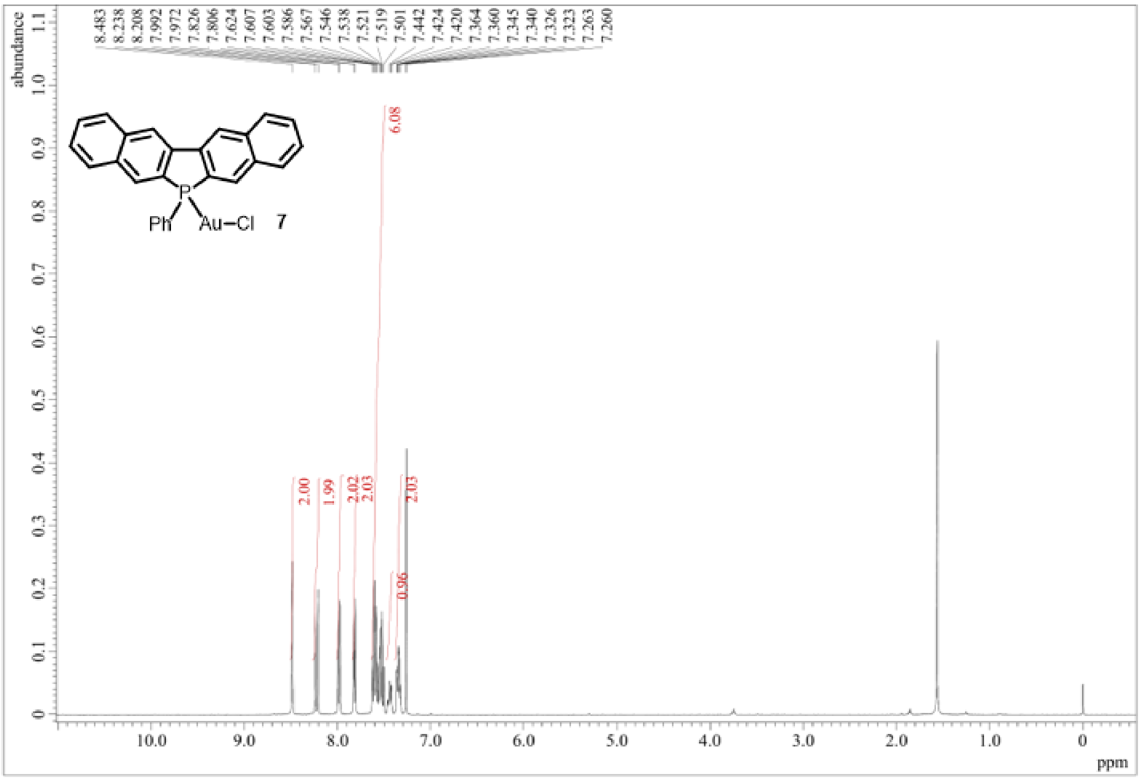

^{13}C NMR spectrum of **7**

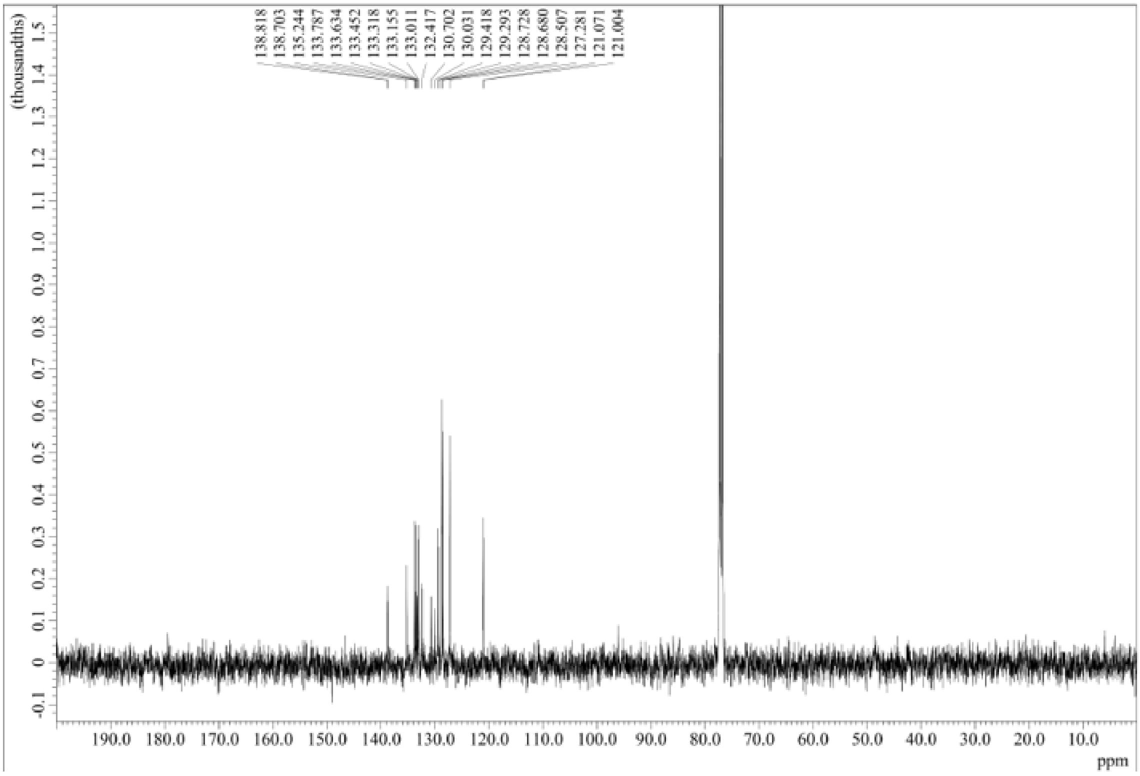

Supplement: File 1 — Further analytical and experimental data. [file Beilstein_J_Org_Chem-17-671-s001.pdf]
